# Zoledronic acid in the treatment of severe scoliosis associated with neurofibromatosis type 1 (NF1): a case report

**DOI:** 10.1097/MS9.0000000000005198

**Published:** 2026-06-11

**Authors:** Changsheng Huang, Shijun Wang, Lei Yue, Haolin Sun

**Affiliations:** Department of Orthopedics, Peking University First Hospital, Beijing, China

**Keywords:** bone loss, case report, neurofibromatosis type 1, scoliosis, zoledronic acid

## Abstract

**Introduction and importance:**

We report the first case of perioperative zoledronic acid administration in an adolescent with neurofibromatosis type 1 (NF1) complicated by severe scoliosis. This case provides a hypothesis-generating experience for bone turnover screening and bisphosphonate intervention in the management of NF1-related scoliosis.

**Presentation of case:**

First case report: adolescent NF1 with severe scoliosis treated by perioperative zoledronic acid plus halo-pelvic traction and spinal fusion. The regimen included preoperative daily traction and 4 mg zoledronic acid, followed by postoperative spinal fusion and 1 mg zoledronic acid. Traction improved spinal alignment and pulmonary function. Bone turnover markers decreased after zoledronic acid administration.

**Clinical discussion:**

For the first time, zoledronic acid was administered perioperatively during primary spinal fusion in an adolescent patient with NF1. Bone turnover markers were reduced after zoledronic acid administration, and a temporal decrease in bone turnover was observed during traction and after surgery. The combination of traction and zoledronic acid was associated with favorable bone marker changes in this patient. A preliminary, hypothesis-generating bone management algorithm is proposed based on this single case: screen for bone markers and spinal CT in patients with severe NF1-associated scoliosis; if high bone turnover or low CT values are detected, consider zoledronic acid administration prior to traction.

**Conclusion:**

In this adolescent patient with NF1-associated severe scoliosis, perioperative zoledronic acid combined with traction and spinal fusion was associated with reduced bone turnover markers and stable implant fixation at a 12-month follow-up. This strategy may provide a hypothesis-generating option for perioperative bone health management. Further large, controlled studies are needed to confirm its efficacy and safety.

## Introduction

Neurofibromatosis type 1 (NF1) is an inherited tumor-predisposing syndrome caused by heterozygous mutations in the NF1 gene[1]. Clinically, it often presents with neurological and cutaneous symptoms, and some patients may also have soft tissue or skeletal abnormalities[2]. Scoliosis is a typical and severe skeletal complication of NF1, which may occur alone or in combination with structural abnormalities such as sharp angulation of 4–6 vertebral bodies, vertebral rotation, vertebral scalloping, vertebral wedging, and rib penciling^[^3,4^]^. Dural ectasia, a major non-tumorous soft tissue manifestation of NF1, usually requires no intervention but may lead to erosion of surrounding bones and vertebral instability[5].

HIGHLIGHTS

- This is the first time zoledronic acid has been used as a perioperative adjuvant therapy in the initial spinal fusion surgery for adolescent patients with severe scoliosis associated with neurofibromatosis type 1 (NF1).
- Zoledronic acid can prevent bone loss related to halo-pelvic traction.
- Zoledronic acid can counteract bone loss caused by NF1.
- This case provides a new therapeutic paradigm for severe scoliosis associated with NF1, which helps optimize intraoperative conditions and reduce surgical risks.

NF1-related scoliosis progresses rapidly and is prone to causing neurological deficits, pain, deformity, and functional disability, necessitating early intervention. Surgery (including growing rod surgery and spinal fusion surgery) is the main treatment method[4]. However, the incidence of complications following spinal surgery in NF1 patients is relatively high, with complication rates of 58.2% for growing rod surgery and 23.3% for spinal fusion surgery, and implant-related complications are the most common[6].

Compared with healthy individuals, NF1 patients generally have reduced bone mass, which can easily progress to osteoporosis[7]. Studies have shown that NF1 patients have lower trabecular bone scores, bone mineralization levels, and bone mineral density (BMD), which are closely associated with severe spinal osteoporosis[8]. At the molecular level, the loss of neurofibromin function leads to abnormal proliferation of osteoclasts, as well as impaired osteoblast activity and collagen production[9], increasing the risk of poor postoperative bone healing and implant loosening.

Preoperative traction is commonly implemented for severe spinal deformity to enhance spinal flexibility and improve surgical safety. However, accumulating evidence has demonstrated that halo-pelvic traction is associated with a significant reduction in BMD, and a positive correlation exists between the magnitude of deformity correction and the degree of BMD loss^[^10,11^]^. Given these findings, additional investigations are warranted in high-risk patient populations to optimize perioperative treatment protocols and develop effective strategies for preventing traction-induced bone loss.

Bisphosphonates (BPs) are commonly used drugs in the clinical treatment of osteoporosis and have been proven to prevent fractures and increase BMD in the long term. As a potent BP, zoledronic acid can bind to hydroxyapatite crystals on the surface of the bone matrix, thereby inhibiting bone resorption and increasing BMD[12]. Animal experiments have shown that zoledronic acid can reduce the ratio of osteoclast surface to bone surface in spinal fusion tissue of NF1 mice, confirming its potential as an adjuvant therapeutic agent to improve the efficacy of spinal fusion in NF1^[^13,14^]^.

This article reports on the treatment process of an adolescent patient with NF1 complicated by severe scoliosis, who underwent spinal fusion after 2 months of halo-pelvic traction with combined perioperative zoledronic acid therapy. It aims to provide a preliminary clinical reference for the perioperative bone management of NF1-related scoliosis.

This case report has been presented in line with the SCARE checklist[15].

## Case report

### Basic information of the patient

A 16-year-old female patient was admitted to the hospital with the complaint of “scoliosis detected for 4 years, accompanied by decreased exercise tolerance and shortness of breath for 1 year.” She had no history of other diseases. After admission, she was diagnosed with NF1 complicated by scoliosis through genetic testing and clinical sign evaluation. Physical examination revealed obvious trunk asymmetry, chest deformity, and weakened breath sounds on lung auscultation. Pulmonary function tests indicated restrictive ventilatory dysfunction, which was considered to be caused by pulmonary compression due to scoliosis.

### Treatment decision-making and implementation

#### Preoperative evaluation and intervention

Preoperative imaging examinations showed that the patient had severe spinal scoliosis and kyphosis deformity, combined with vertebral rotation, vertebral scalloping, vertebral wedging, and rib penciling, with obvious pulmonary compression (Fig. 1). CT examination indicated severe dural ectasia in the entire thoracolumbar spine, which was most prominent in the T5–T10 segment (Fig. 2). Given the severe spinal deformity of the patient, direct surgical correction was difficult and risky. Therefore, the clinical team decided to first perform halo-pelvic traction to improve spinal alignment and reduce pulmonary compression.

**Figure 1.**
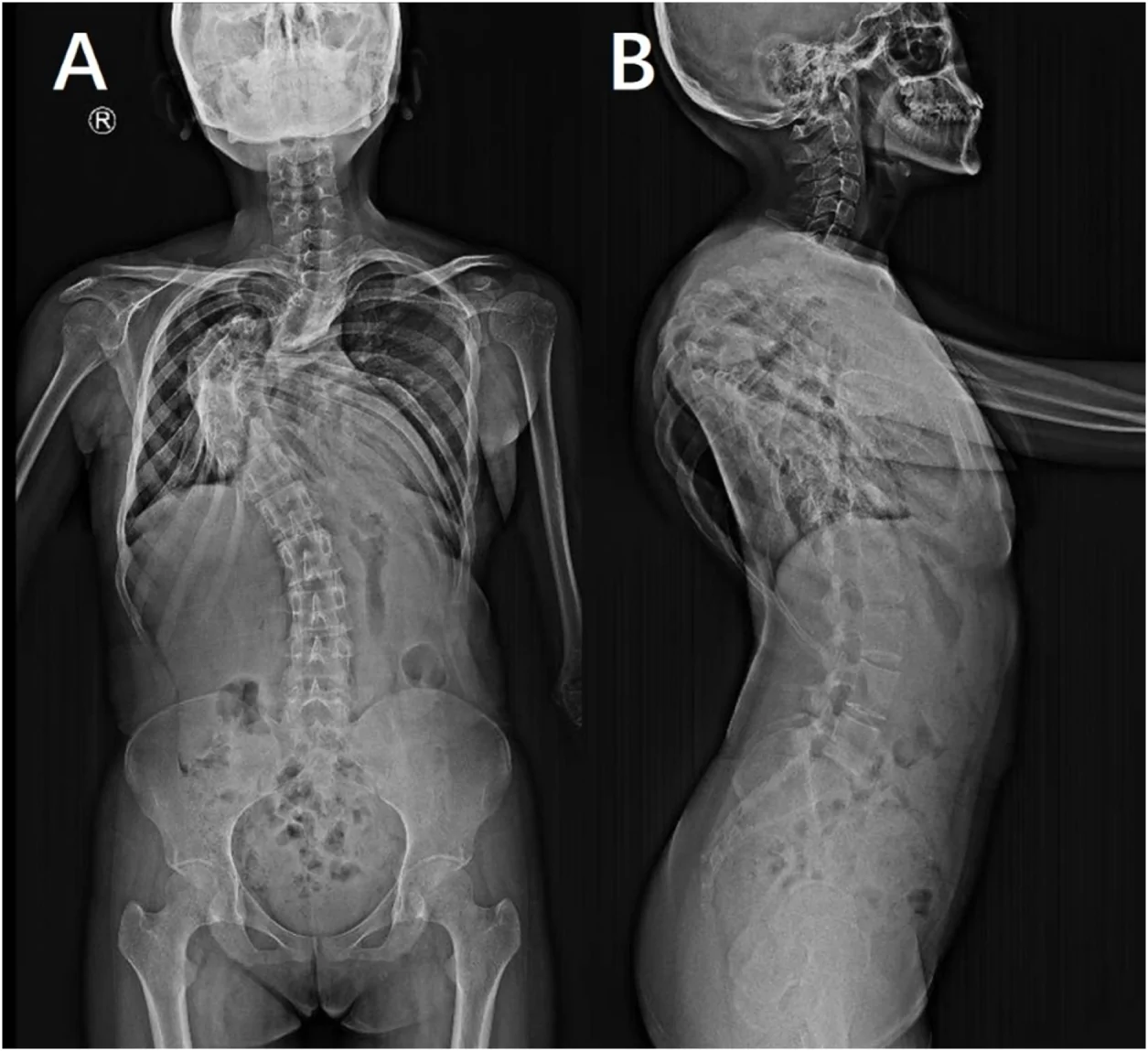
Full-spine X-ray of a 16-year-old female, showing severe scoliosis and kyphosis deformity, combined with vertebral rotation, vertebral scalloping, vertebral wedging, and rib penciling, mainly involving the thoracic spine. Obvious pulmonary compression is also visible in the patient.

**Figure 2.**
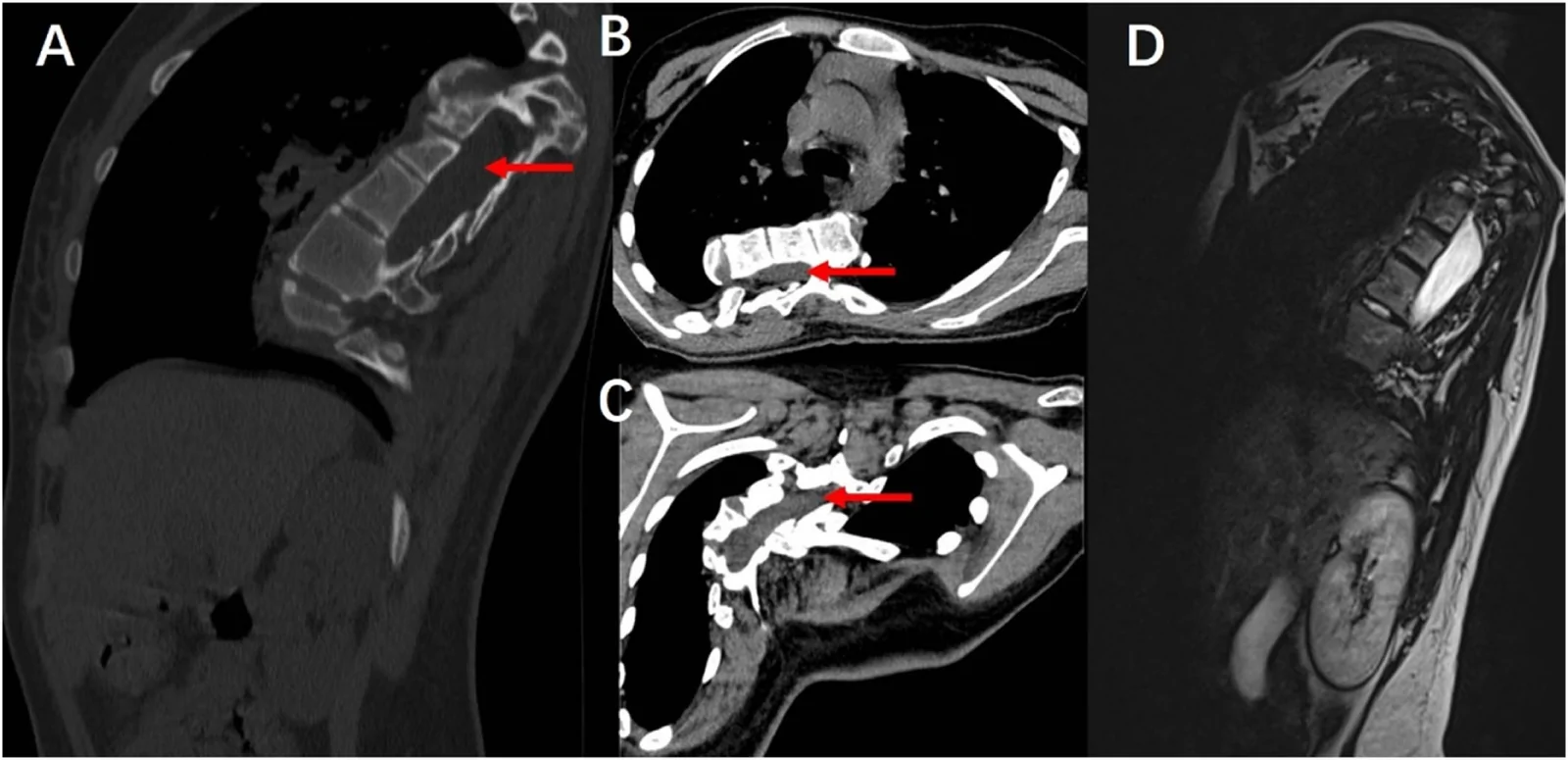
(A–C) Thoracic spine CT of a 16-year-old female, showing severe dural ectasia throughout the entire thoracic spine, along with obvious vertebral scalloping and a kyphosis deformity, which is most severe in the T5–T10 segment. (D) Thoracic spine MRI of a 16-year-old female, showing an S-shaped scoliosis of the spine, an intact spinal canal structure, and dural ectasia.

Meanwhile, considering that NF1 patients are prone to bone loss, preoperative detection of bone metabolism markers was conducted: type I collagen amino-terminal propeptide (PINP) was 128.8 ng/ml (reference range for premenopausal women: 15.13–58.59 ng/ml), and β-collagen degradation product (β-CTX) was 1.68 ng/ml (reference range for premenopausal women: ≤ 0.573 ng/ml). Both indicators were higher than the normal range, suggesting that the patient was in a state of high bone loss. The patient was 16 years old. No age-specific reference ranges for children and adolescents were available, which limited data interpretation. However, relevant studies have found that the normal values of bone metabolism indicators in 16-year-old children are not different from those of adults[16]. Zoledronic acid (4 mg intravenous infusion) was administered to modulate bone metabolism on 27 May 2024.

#### Efficacy of halo-pelvic traction

Halo-pelvic traction was continued for 2 months, with a daily height adjustment of 2–3 mm under close monitoring of neurological status and pin tract conditions. The height increment was reduced if pain occurred, based on the patient’s response. No pin tract infection, cranial nerve palsy, or neurological deficit was observed during traction. Full-spine X-ray films were regularly rechecked during the traction period (Fig. 3). The results showed that the degree of spinal scoliosis gradually decreased after 3, 5, and 7 weeks of traction, and her height increased by 12 cm compared with that before traction. Pulmonary function re-examination indicated improved ventilatory dysfunction and alleviated pulmonary compression symptoms.

**Figure 3.**
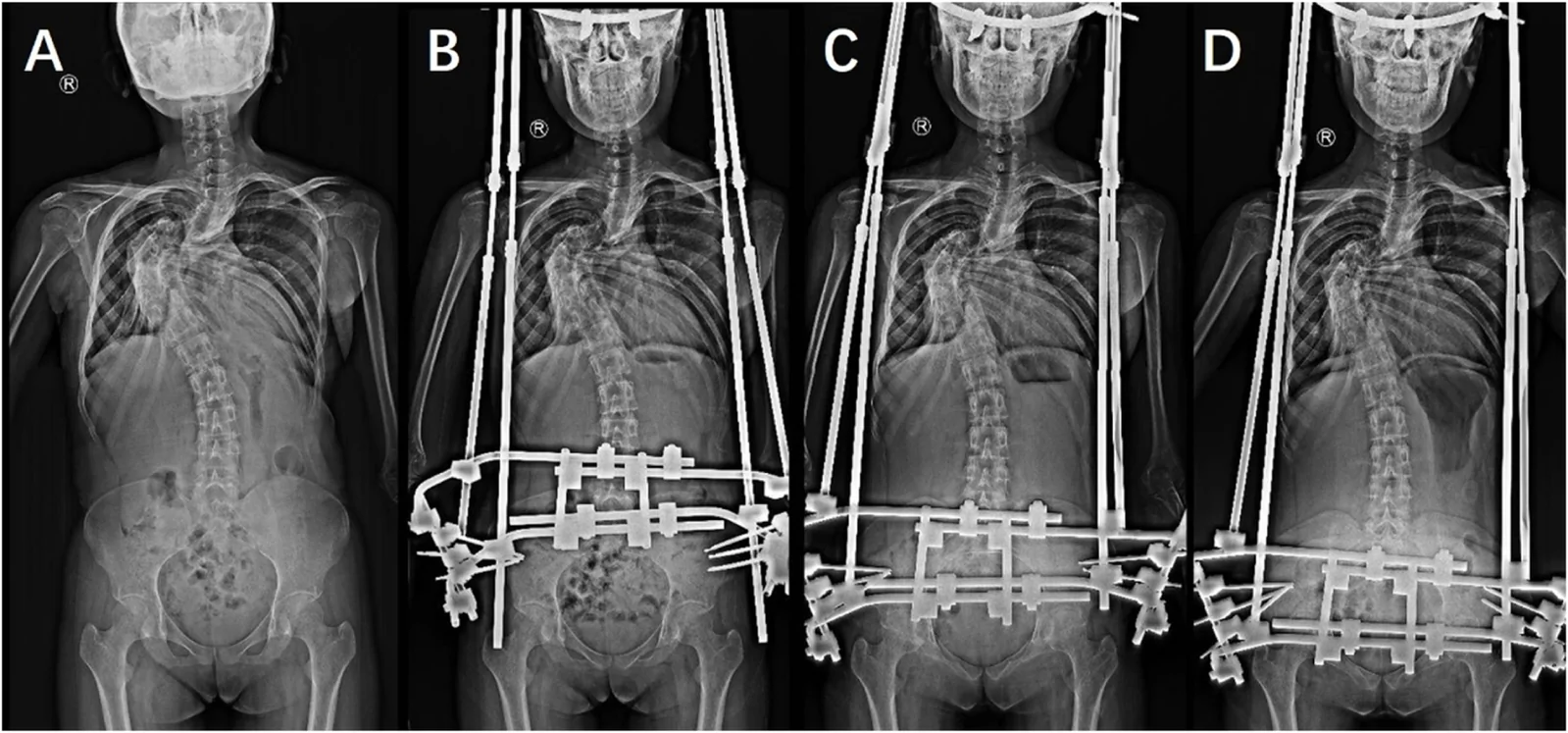
(A) Full-spine X-ray of a 16-year-old female before halo-pelvic traction. (B–D) Full-spine X-rays of the patient at 3 weeks, 5 weeks, and 7 weeks after halo-pelvic traction, respectively. Compared to the condition before halo-pelvic traction, the degree of scoliosis in the patient gradually decreased, her height gradually increased, and the pulmonary compression was slightly alleviated.

Bone metabolism markers were monitored regularly, and PINP and β-CTX showed a gradual downward trend. By the end of traction on 1 July 2024, they had decreased to 51.77 ng/ml and 0.36 ng/ml, respectively, indicating a reduction in bone turnover markers following zoledronic acid administration. The intact parathyroid hormone (iPTH) increased from 62.57 pg/ml (reference range: 15–65 pg/ml) on 22 May 2024 to 84.25 pg/ml on 12 June 2024, and then decreased to 61.18 pg/ml at the end of traction. 25-Hydroxyvitamin D (25-OH-VitD) increased from 36 nmol/L on 22 May 2024 to 37.2 nmol/L on 12 June 2024, and further increased to 54.8 nmol/L on 1 July 2024, showing a fluctuating upward trend, which indicated the gradual improvement of the body’s vitamin D nutritional status and calcium regulation mechanism during treatment.

#### Spinal surgery treatment

On 18 July 2024, 2 months after traction, the patient underwent spinal scoliosis correction surgery + spinal fusion surgery + thoracoplasty. The operation was performed smoothly, and spinal internal fixation devices (screws and rod systems) were placed intraoperatively to maintain the effect of spinal correction. Immediate postoperative evaluation showed that the sensory and motor functions of the upper limbs were normal; however, the muscle strength of the lower limbs decreased to grade 3, which was considered to be caused by intraoperative traction. Symptomatic treatments, such as neurotrophic therapy and rehabilitation training, were administered, and the muscle strength of the lower limbs recovered to grade 5 after 10 days.

#### Postoperative intervention and follow-up

Two weeks after surgery, re-examination of bone metabolic markers revealed an increase in PINP to 192.4 ng/ml and β-CTX to 0.86 ng/ml, indicating that major spinal surgery stimulated bone metabolism and accelerated bone turnover. On 9 August 2024, zoledronic acid (1 mg via intravenous infusion) was administered again to modulate postoperative bone resorption. The patient tolerated both infusions well; only acute fever developed 2 days after the first injection, which resolved with physical cooling, and no other adverse reactions were observed.

During the subsequent follow-up, re-examination on 21 October 2024 showed that full-spine X-rays revealed a good effect of spinal scoliosis correction, no loosening or fracture of the internal fixation devices, and a significant improvement in the overall spinal curve compared with that before the operation (Fig. 4). Pulmonary imaging examinations indicated that the pulmonary compression was completely relieved, and the lung tissue was significantly expanded. Re-examination of bone metabolism markers showed that PINP decreased to 112.4 ng/ml (close to the level at admission), β-CTX decreased to 0.49 ng/ml (returning to the normal range), iPTH further decreased from 61.18 pg/ml after the operation to 25.15 pg/ml, and 25-OH-VitD was maintained at 56.5 nmol/L.

**Figure 4.**
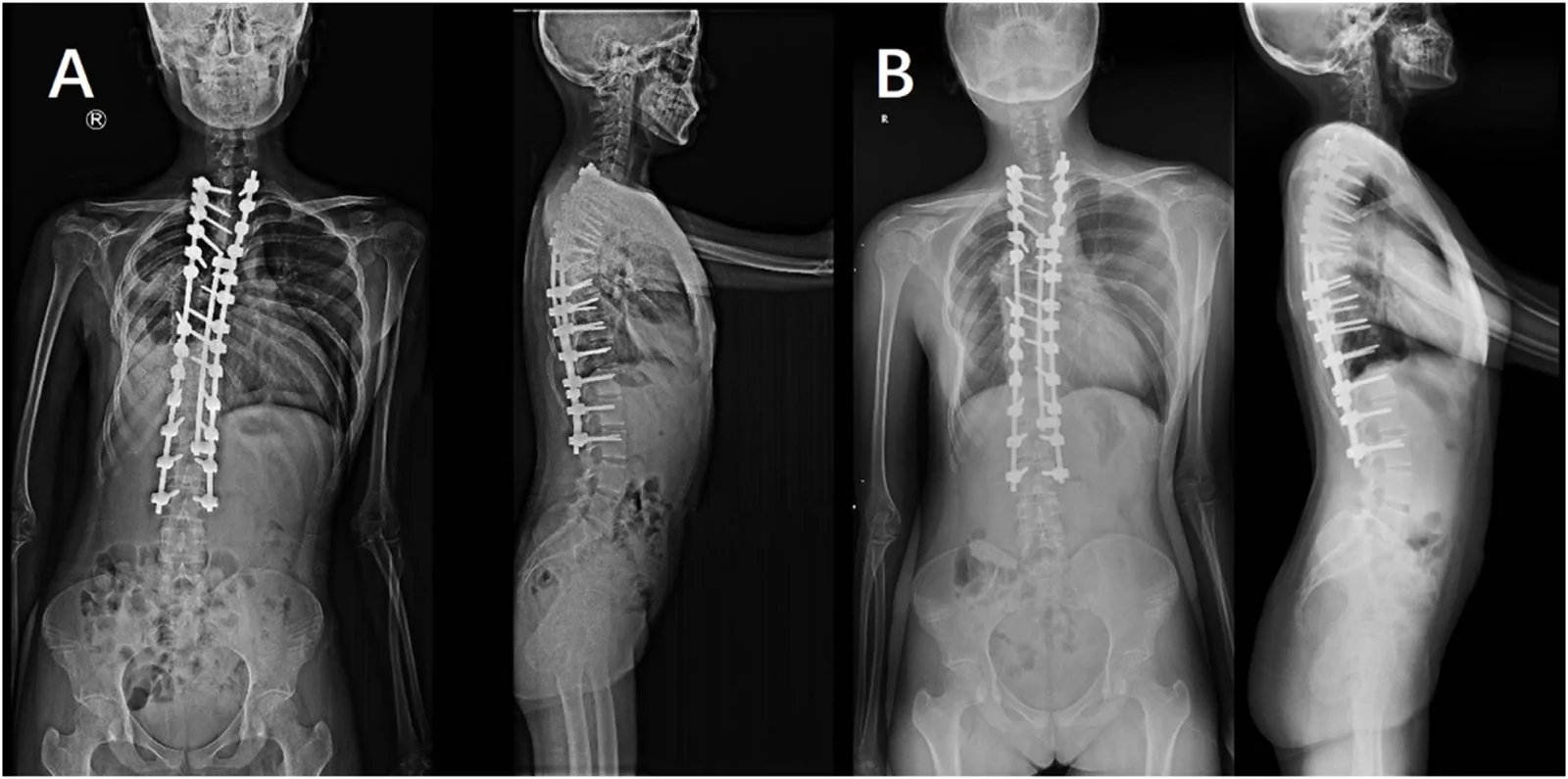
(A) Full-spine X-ray of a 16-year-old female, taken 3 weeks after spinal correction surgery, showing that the spinal scoliosis and kyphosis deformity were significantly improved compared with those before the operation. The compression of the right lung was slightly improved, and there was no obvious loosening of the internal fixation devices in the body. (B) Full-spine X-ray of a 16-year-old female, taken 3 months after spinal correction surgery, showing that the pulmonary compression was relieved, the lungs were significantly expanded, and there was no loosening or fracture of the internal fixation devices in the body compared with the previous state.

In the latest re-examination on 29 July 2025 (12 months postoperatively), iPTH was 19.59 pg/ml (within the normal reference range), indicating stable parathyroid function; 25-OH-VitD increased to 59.9 nmol/L, reflecting continuous optimization of vitamin D levels; PINP decreased to 68.6 ng/ml, β-CTX decreased to 0.35 ng/ml, and osteocalcin increased from 16.48 ng/ml on 21 October 2024 to 18.83 ng/ml. Various bone metabolism markers continued to improve, the internal fixation devices remained stable, the effect of spinal scoliosis correction was maintained, and pulmonary function was normal. These observations suggest a temporal association between perioperative zoledronic acid and favorable bone metabolic changes in this patient. All bone metabolism markers of the patient during the study period can be seen in Table 1.

**Table 1:** Bone metabolism markers of the patient during the study period.

	22 May 2024	27 May 2024	12 June 2024	01 July 2024	18 July 2024	9 August 2024	21 October 2024	29 July 2025
Spinal fusion surgery	-	-	-	-	X	-	-	-
Zoledronic acid	-	XX	-	-	-	XX	-	-
iPTH, pg/ml	62.57	-	84.25	61.18	-	38.66	25.15	19.59
25-OH-VitD, nmol/l	36	-	37.2	54.8	-	36	56.5	59.9
PINP, ng/ml	128.8	-	101.1	51.77	-	192.4	112.4	68.6
β-CTX, ng/ml	1.68	-	0.49	0.36	-	0.86	0.49	0.35
OC, ng/ml	37.88	-	33.67	15.36	-	15.09	16.48	18.83

X: indicates the performance of spinal fusion surgery.

XX: indicates administration of zoledronic acid.

Intact parathyroid hormone (iPTH): Reference range is 15–65 pg/ml.

25-Hydroxyvitamin D (25-OH-VitD): sufficient: 75–250 nmol/l; insufficient: 25–74 nmol/l; deficient: <25 nmol/l; and potential toxicity: >250 nmol/l.

Procollagen type I N-terminal propeptide (PINP): premenopausal: 15.13–58.59 ng/ml; postmenopausal [with hormone replacement therapy (HRT)]: 14.28–58.92 ng/ml; and postmenopausal (without HRT): 20.25–76.31 ng/ml.

Beta-crossLaps of type I collagen (β-CTX): premenopausal: ≤0.573 ng/ml; postmenopausal: ≤1.008 ng/ml.

Osteocalcin (OC): premenopausal: 11–43 ng/ml; postmenopausal: 15–46 ng/ml.

#### Imaging evaluation

For patients with severe scoliosis, BMD measurement at the lumbar spine is less accurate than at the hip joint due to spinal deformity. Additionally, in the patient of this study, after halo-pelvic traction, metal screws on the ilium and the traction frame caused artifacts that interfered with BMD measurement accuracy. To accurately assess the patient’s BMD, lumbar spine CT scans were performed at key time points throughout the treatment course, and the CT values of the L1–L5 vertebrae were measured[17]. Hounsfield units (HU) from CT were used as a surrogate marker for BMD, derived from a clinical study via a linear regression equation: HU = 122 + 15.04 × lumbar spine *T*-score[18].

CT HU values were used as a supportive surrogate marker for bone mass status, not as direct proof of BMD recovery. It should be noted that CT HU, as a surrogate for BMD, has certain limitations in this study: first, the severe spinal scoliosis and vertebral structural abnormalities (scalloping and wedging) of the NF1 patient may lead to uneven vertebral bone density distribution, which affects the representativeness of the mean HU value of L1–L5; second, the metal artifacts from traction devices and internal fixation may cause partial pixel distortion of CT images, and we minimized this interference by selecting the vertebral body areas without obvious artifact overlap for measurement; third, HU values are affected by CT scanning parameters (e.g., tube voltage and slice thickness), and all scans in this study were completed on the same device with unified parameters to ensure the comparability of HU values at different time points.

Preoperative measurement (16 May 2024) showed a mean CT value of 167.62 HU for the L1–L5 vertebrae, which, combined with abnormal bone metabolic markers, suggested insufficient bone mass reserve. Re-examination at the end of halo-pelvic traction (12 July 2024) revealed a mean CT value of 140.42 HU for the L1–L5 vertebrae, indicating mild bone loss during traction. In the early postoperative period (22 July 2024), the mean CT value recovered to 170.00 HU. Long-term postoperative follow-up (28 July 2025) demonstrated that the mean CT value increased to 174.19 HU. No failure of internal fixation devices was observed, and imaging findings at 12 months were consistent with progressing fusion (Fig. 5).

**Figure 5.**
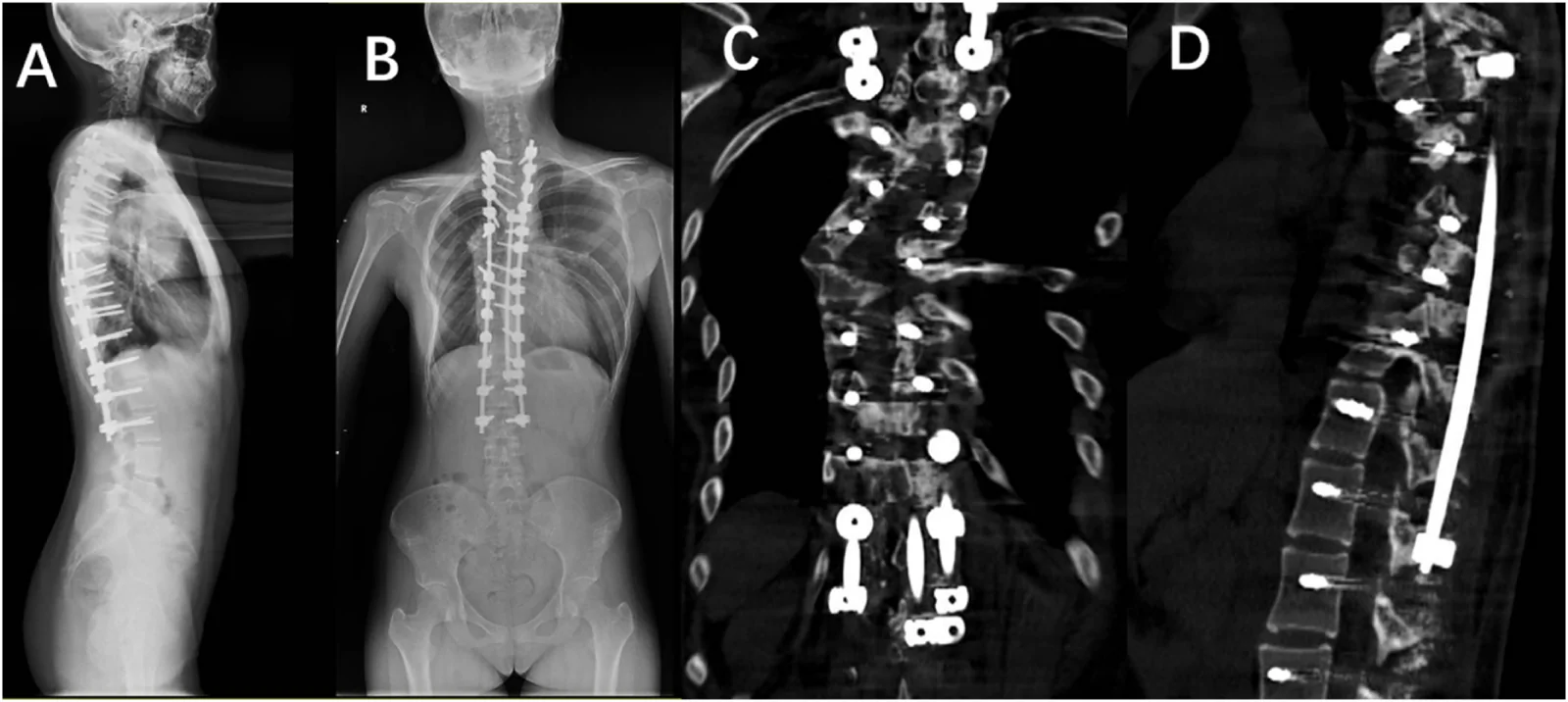
(A and B) Full-spine X-rays taken 1 year after the operation, showing no fractures of the internal fixation devices in the patient’s body. (C and D) Sagittal and coronal views of the spinal CT taken 1 year after the operation, showing obvious bone fusion in the surgical area of the patient (especially in the area with severe deformity).

Note: ROIs were selected in the vertebral body without obvious artifact overlap; endplate and cortical bone were excluded. All measurements were independently performed by two observers in duplicate, and the mean values were used for analysis. Severe deformity, vertebral scalloping, wedging, traction hardware, and postoperative implants may affect HU reproducibility.

The patient was 16 years old. All reference ranges shown are for adult premenopausal women; age-appropriate pediatric/adolescent reference ranges were not available. The interpretation of “normal” or “abnormal” is limited by the use of adult reference values.

#### Patient perspective and ethical considerations

Written informed consent for treatment and the publication of this case report was obtained from the patient and her legal guardians. They expressed satisfaction with the treatment outcomes, particularly the relief of respiratory symptoms and improvement in posture, and understood that the off-label use of zoledronic acid was part of a comprehensive bone management strategy for this severe case.

During treatment, the patient received vitamin D and calcium supplementation, nutritional support, and progressive mobilization as part of routine perioperative care. These interventions may have contributed to the observed changes in bone metabolism markers and HU values.

## Discussion

While zoledronic acid has been previously reported in spinal fusion revision surgery for adult NF1 patients with scoliosis and established osteoporosis[19]. To our knowledge, this is the first report of perioperative zoledronic acid administration during primary spinal fusion in an adolescent NF1 patient, implemented with a preventive intent to mitigate both NF1-related and traction-induced high bone turnover. The preoperative dose of 4 mg (≈0.1 mg/kg) was selected based on pediatric BP guidelines, and the postoperative 1 mg dose was administered to maintain annual cumulative exposure below 5 mg[20]. Only a self-limited acute-phase reaction was observed, underscoring the favorable short-term safety profile of zoledronic acid in this adolescent patient.

Adolescent NF1 patients are at a critical stage of peak bone mass acquisition; bone loss during this period may compromise lifelong skeletal health[6]. In this case, zoledronic acid administration was temporally associated with decreased bone turnover markers. Correspondingly, the mean lumbar CT value recovered from 140.42 HU after traction to 174.19 HU at the 1-year follow-up, with no implant loosening and evident fusion mass formation. These single-case findings suggest that early, proactive bone modulation may reduce the potential risk of nonunion and implant-related complications in NF1-associated scoliosis surgery, and the short-term efficacy of perioperative zoledronic acid for improving bone metabolic status is supported by the longitudinal bone marker and HU value data.

A particular clinical dilemma in severe NF1-related deformity is the necessity of preoperative halo-pelvic traction to improve spinal flexibility and reduce surgical risk, despite its known adverse effect on BMD^[^10,11^]^. Our case demonstrates that pre-traction administration of zoledronic acid was associated with attenuation of bone turnover during traction, which may help to balance the benefits of traction and skeletal safety in this patient.

Studies have shown that both adult and pediatric NF1 patients have lower BMD than healthy individuals[21], and the levels of bone loss-related indicators such as serum parathyroid hormone, PINP, and CTX are increased[22]. At the molecular level, the loss of neurofibromin not only leads to defects in osteoblast differentiation[23] but also enhances the osteolytic activity of osteoclasts[24]. These dual factors accelerate bone loss. Therefore, taking early measures to inhibit osteoclasts and slow down bone loss is the key to preventing NF1-related orthopedic complications[25]. In this case, the 16-year-old patient had already developed obvious bone loss (increased PINP and β-CTX). Preoperative application of zoledronic acid could improve bone conditions in advance, lay a foundation for spinal fusion surgery, and reduce the risk of postoperative nonunion.

NF1-related scoliosis progresses rapidly, and conservative treatment and brace treatment cannot delay its progression[4], thus requiring early surgical intervention. However, direct surgery for patients with severe scoliosis is associated with high risks, such as nerve injury and aggravated pulmonary compression. In this case, 2 months of halo-pelvic traction increased the patient’s height by 12 cm and improved pulmonary function, which not only reduced the difficulty of surgery but also created conditions for postoperative pulmonary function recovery, consistent with previous studies on halo ring traction as an adjuvant for severe NF1 spinal deformity surgery^[^26,27^]^.

Perioperative zoledronic acid application was temporally associated with changes in bone turnover markers in this patient: preoperative administration coincided with reduced bone turnover during traction, and postoperative administration coincided with mitigation of the postoperative bone turnover peak. Zoledronic acid did not significantly change the levels of iPTH and 25-OH-VitD in the short term (within 2 weeks), but significant improvements in both indicators were observed at the 1-year follow-up, suggesting that the regulation of bone metabolism by zoledronic acid is time-dependent, and long-term monitoring is required to evaluate its sustained effect.

Based on this single-case experience, we propose a preliminary, hypothesis-generating bone management protocol for adolescents with NF1 complicated by severe scoliosis who are scheduled for spinal fusion surgery: (1) pre-traction stage: Screen for serum PINP/β-CTX and perform lumbar CT; if dual-emission X-ray absorptiometry (DXA) is not feasible, measure the HU value to evaluate bone mass status with strict quality control (unified scanning parameters and artifact avoidance). (2) If the patient presents with high bone turnover (PINP or β-CTX level exceeding the upper limit of normal) or low bone mass indicated by CT HU value, consider a single dose of zoledronic acid (0.1 mg/kg) before traction. (3) Implement halo-pelvic traction with gradual weight increase and close monitoring throughout the process. (4) Post-fusion stage: Re-examine bone turnover markers 2–4 weeks after surgery; if a significant elevation is observed, consider administering a second dose of zoledronic acid. (5) Conduct long-term follow-up with annual bone marker testing and imaging examinations. This protocol is hypothetical and requires validation in larger patient cohorts and prospective studies.

This study has several inherent limitations due to its single-case design. Firstly, the small sample size cannot fully verify the universality of this treatment plan, and causal inferences regarding the reduction of postoperative complications cannot be established without a control group. Secondly, the follow-up time was only 1 year, making it difficult to comprehensively evaluate the long-term efficacy and safety of zoledronic acid in adolescent NF1 patients. Thirdly, the optimal dosage, cycle, and timing of zoledronic acid for NF1 patients have not yet been clarified, and more clinical studies are needed for exploration. Fourthly, long-term use of zoledronic acid may be associated with side effects such as decreased efficacy and osteonecrosis of the jaw, so long-term follow-up and monitoring of patients are required. Finally, only five bone metabolism markers were detected in this case; in the future, more laboratory examinations (such as DXA when feasible and comprehensive detection of bone turnover markers) need to be conducted to more comprehensively evaluate the patient’s bone metabolism status.

## Conclusion

This single case report demonstrates that, for adolescents with NF1 complicated by severe scoliosis, perioperative administration of zoledronic acid combined with halo-pelvic traction and spinal fusion surgery was temporally associated with reduced bone turnover markers and stable implant fixation at a 12-month follow-up.

This approach provides a hypothesis-generating perspective for perioperative bone health management. Due to the limitations of a single-case study, no causal efficacy can be confirmed, and further controlled studies are needed.

## Ethical approval

The study was conducted in accordance with the Declaration of Helsinki and was approved by the Biomedical Ethics Committee of Peking University First Hospital (Approval no: 2022-744).

## Data Availability

The original contributions presented in this study are included in the article. Further inquiries may be directed to the corresponding author.

## Research registration unique identifying number (UIN)

Not applicable (single case report in compliance with the SCARE 2025 guidelines).

## Presentation statement

This is an original case report that has not been presented at any conference or published elsewhere.
